# Secondary invasion re‐redefined: The distinction between invader‐facilitated and invader‐contingent invasions as subclasses of secondary invasion

**DOI:** 10.1002/ece3.3966

**Published:** 2018-04-27

**Authors:** Dean E. Pearson, Yvette K. Ortega, Justin Runyon, Jack L. Butler

**Affiliations:** ^1^ Rocky Mountain Research Station USDA Forest Service Missoula MT USA; ^2^ Division of Biological Sciences University of Montana Missoula MT USA; ^3^ Rocky Mountain Research Station USDA Forest Service Bozeman MT USA; ^4^ Rocky Mountain Research Station USDA Forest Service Rapid City SD USA

**Keywords:** biological invasion, reinvasion, secondary invasion, weed management, weed treadmill

## Abstract

**Linked Article**: https://doi.org/10.1002/ece3.3964

In their recent article in *Ecology and Evolution*, O'Loughlin and Green (2017) set out to (1) redefine the term secondary invasion as the condition “when invader success is contingent on other invaders altering the properties of recipient ecosystems” and (2) propose a framework for accounting for this phenomenon in invasion ecology. We applaud the second objective. However, redefining the term secondary invasion in the very narrow manner they propose is problematic and unfounded. Here, we establish that secondary invasion represents a broad range of phenomena that encompasses invader‐facilitated invasions of which the condition described by O'Loughlin and Green, invader‐contingent invasions, is a special case. We further demonstrate how recognizing these distinctions and applying these definitions in this manner expands the applicability of the framework they propose for thinking about facilitation in invasion ecology.

O'Loughlin and Green based their definition of secondary invasion on precedence. They reviewed the use of “secondary invasion” based on a literature search for this term in titles, abstracts, or keywords of ecologically oriented scientific publications and stated that “The first usage of the term in a broadly ecological context was by Wicklow, Bennett, and Shotwell (1987) to describe plant–pathogen dynamics in soybeans, where one fungal pathogen could only affect crops already infected by a different fungal pathogen (Wicklow, Bennett, & Shotwell, 1987).” Based on this precedence, they advocate for “the very narrow use of the term” as “the phenomenon in which invasion success of one exotic species (the secondary invader) is completely contingent on the presence, influence and impact of one or more other exotic species (primary invaders).” Firstly, precedence alone is not sufficient justification for defining scientific nomenclature (e.g., Gould & Vrba, 1982). Secondly, the approach they took in establishing the precedence of usage was arbitrary as the term secondary invasion has been used by many authors prior to Wicklow, Bennett, and Shotwell (1987) in a variety of ecological contexts. A simple Google Scholar search for published papers that use the terms “ecology” and “secondary invasion” anywhere in the article demonstrates that secondary invasion has been used hundreds of times up to the publication of Wicklow, Bennett, and Shotwell (1987) and in a broader range of ecological contexts than that outlined by O'Loughlin and Green in their table 1. The most common usage up to 1987 aside from that of Wicklow, Bennett, and Shotwell (1987) is its application to successional ecology (e.g., Bormann, 1953; Brown, 1953; Carleton & Maycock, 1978; Faegri, 1937; Humphrey, 1958; Kormondy, 1969; Michelmore, 1939).

While the usage of this term in disease ecology is certainly legitimate and could be arguably applied to invasion ecology in the manner proposed by O'Loughlin and Green, we suggest that its usage in successional ecology provides a more fitting parallel for its application to invasion ecology because of the broader functionality of the term and range of underlying processes addressed. In the context of successional ecology, secondary invasion has been used in reference to the sequence of arrival of newcomers within and among successional seres. In succession theory, sequential arrival of new,comers is most often not obligatorily facilitated by prior arrivals (sensu Gleason, 1926), although of course, prior arrivals may alter conditions in ways that do facilitate later arrivals (sensu Clements, 1916) and in some cases, such processes may involve obligatory facilitation (e.g., Turner, 1983), although this is probably not common (Connell & Slatyer, 1977). These ideas provide direct parallels to facilitation in invasion ecology as outlined below.

O'Loughlin and Green's Venn diagram in figure 2 and community interaction web in figure 3 layout pathways by which a primary invader may facilitate other invaders by altering components of the recipient ecosystem in ways that benefit the secondary invader or by providing new ecosystem components that directly benefit the secondary invader. This framework is certainly valuable for thinking about how some invaders may facilitate other invaders. However, the processes and value of the framework are by no means limited to the type of obligatory facilitation emphasized by the authors, wherein secondary invasion is “completely contingent” on the primary invader. In fact, the obligatory facilitation that they describe is likely such a rare case of invader‐facilitated invasion that applying this framework to only this situation negates the broader value of the ideas proposed. The authors provide many examples of invader‐facilitated invasions in describing their framework, but their review generated only two cases that may actually meet the stringent criteria of secondary invaders being obligatorily facilitated by a primary invader (i.e., Grosholz et al., 2000; O'Dowd, Green, & Lake, 2003). While many invaders may facilitate secondary invasions and invaders that are strong mutualists and ecosystem engineers may in some cases open doors to secondary invaders that were otherwise so tightly closed as to preclude entry (e.g., Grosholz et al., 2000; O'Dowd et al., 2003), the bulk of the cases of invader‐facilitated invasions are arguably examples of primary invaders (or even concurrent or later invaders) facilitating other invaders that were not excluded from the system (e.g., Flory & Bauer, 2014; see also examples in Kuebbing & Nuñez, 2015; O'Loughlin & Green, 2017; Simberloff, 2006; Simberloff & Von Holle, 1999; White, Wilson, & Clarke, 2006). A general finding from invasion ecology is that most communities are open to invasion (Callaway & Maron, 2006), and there are surprisingly few examples of invaders that are physiologically capable of establishing in a system but are otherwise completely excluded (Mack, 1996).

The fact that obligatory facilitation may be rare is no reason to ignore it, and we agree that highlighting this process may inspire further study that unveils additional interesting and informative examples. However, co‐opting a generic term that explains a range of processes for the purpose of explaining one special case of that broader set of processes is counterproductive. Invader‐contingent invasions are a special case of invader‐facilitated invasions. Accordingly, the framework proposed by O'Loughlin and Green is a valuable one if it is applied to account for invader‐facilitated invasions as the more general case where invaders facilitate other invaders that are not otherwise excluded from the system, and it treats invader‐contingent invasions as a special case of the latter. Hence, we suggest that the terms invader‐facilitated invasion and invader‐contingent invasion be adopted to describe these cases of secondary invasion.

The term secondary invasion has been applied very broadly in ecology to refer to sequential invasion events in successional ecology (e.g., Bormann, 1953; Brown, 1953; Carleton & Maycock, 1978; Faegri, 1937; Kormondy, 1969), evolutionary and paleoecology ecology (e.g., Coulson, Marshall, Pepin, & Carr, 2006; Pramuk, Robertson, Sites, & Noonan, 2008; Vanzolini, 1968), and invasion ecology (e.g., Albaina et al., 2016; Baldwin, Carpenter, Rury, & Woodward, 2012; Dietz & Edwards, 2006; Pearson, Ortega, Runyon, & Butler, 2016; Root, 1964). Its use in disease ecology (e.g., Wicklow, Bennett, & Shotwell, 1987) represents a special case of these broader classes of secondary invasion. We suggest that narrowly defining such a generic term is unwarranted. Many biotic and abiotic factors may influence the sequence of invasion events and allowing for this diverse range of processes is fundamental to their study. Recognizing these broader applications of this term can be readily accounted for by identifying the specific types of secondary invasion being studied, such as invader‐contingent secondary invasion.

## FUNDING INFORMATION

This work was supported by funding from the Rocky Mountain Research Station, USDA Forest Service.

## CONFLICT OF INTEREST

None declared.

## AUTHOR CONTRIBUTIONS

DP developed the initial draft. YK, JR, and JB critically contributed to the draft. All authors gave final approval for publication.
